# HTLV-1 evades Type 1 interferon antiviral signaling by inducing the suppressor of cytokine signaling 1 (SOCS1)

**DOI:** 10.1186/1742-4690-8-S1-A151

**Published:** 2011-06-06

**Authors:** Stéphanie Olière, Eduardo Hernandez, Agnès Lézin, Renée Douville, Raymond Césaire, John Hiscott

**Affiliations:** 1 Molecular Oncology Group, Lady Davis Institute, Jewish General Hospital, McGill University, Montreal, H3T 1E2, Canada; 2 Laboratoire de Virologie-Immunologie, and JE2503, Centre Hospitalier Universitaire de Fort-de-France, 97.2, Martinique; 3 Vaccine and Gene Therapy Institute, Port St. Lucie, Fl, 34987, USA

## 

Although most HTLV-1-infected individuals remain asymptomatic carriers (AC) during their lifetime, 2-5% will develop either Adult T-cell Leukemia (ATL) or the neurological disorder HTLV-1-associated myelopathy/tropical spastic paraparesis (HAM/TSP). To better understand differential gene expression in HTLV-1-associated disease, we examined the mRNA profiles of CD4+ T-cells isolated from ATL, HAM/TSP, AC and non-infected controls. Using genomic approaches, we identified gene signatures that discriminate between HTLV-1 clinical outcomes. Major activated cellular pathways included antimicrobial defense (KLRB1/SPN/SELPLG), innate immune sensing (TRAF3/AIM2/TLR2/TLR4/IKBKG/STAT3), cell adhesion and chemotaxis (CXCR4/CD2/CD63/CD48/CCL14/SPN/CCL13), thus supporting the idea of global immune disruption during HTLV-1 infection. Among the many genes identified, the suppressor of cytokine signaling 1, SOCS1, was upregulated in HAM/TSP and AC. SOCS1 expression positively correlated with high HTLV-1 mRNA load that is characteristic of HAM/TSP patients. SOCS1 inhibited cellular antiviral signaling during HTLV-1 infection by degrading IRF3, an essential transcription factor in the interferon pathway. This novel mechanism of viral evasion of the innate immune response can be directly related to the efficiency of HTLV-1 replication in patients with HAM/TSP. Despite SOCS1 ability to limit antiviral responses, CD4+ T-cells from HAM/TSP patients revealed a striking upregulation of IFN stimulated gene expression, viral host restriction factors and pro-inflammatory genes. We hypothesize that the magnitude of innate immune responses against HTLV-1 during de novo infection may predict clinical outcome; patients mounting a sustained/low antiviral response to HTLV-1 may remain AC with a low probability to progress to ATL, while those displaying an aberrant/high IFN response may progress to HAM/TSP.

